# Improving Health Through Health Marketing

**Published:** 2006-06-15

**Authors:** Jay M Bernhardt

**Affiliations:** National Center for Health Marketing, Coordinating Center for Health Information and Service, Centers for Disease Control and Prevention

Mention the word *marketing* to employees at most companies, and they will understand how it relates to their business. Successful commercial marketing can mean the difference between a company's profit and loss, jobs or layoffs, growth or bankruptcy. Mention the word *marketing* to public health professionals, and you may get blank stares, head shakes, or looks of confusion (and I've seen them all). Marketing is not well-known or understood in the public health community, in part because it is rarely taught in public health or medical schools. Nonetheless, the time has come to increase our awareness, training, and application of health marketing strategies and principles, because the field holds great promise for increasing the adoption of health promotion and protection information and interventions.

## Defining Health Marketing

As we grapple with ways to best reach the public and improve health, principles of commercial marketing are an underused resource. According to the American Marketing Association (1), marketing involves "creating, communicating, and delivering value to customers and for managing customer relationships in ways that *benefit the organization and its stakeholders*" (emphasis added). The Centers for Disease Control and Prevention (CDC) has adapted this definition by defining health marketing as "creating, communicating, and delivering health information and interventions using consumer-centered and science-based strategies to protect and promote the health of diverse populations." Health marketing uses the science and strategies of commercial marketing to promote its products, namely, evidence-based health information and interventions. Although the ultimate goal of commercial marketing is to benefit the product "sellers" and shareholders, the ultimate goal of health marketing is to benefit the product "consumers" and the public.

The science and practice of health marketing draws heavily from several related and often overlapping disciplines and models. Market research, marketing strategy, and public relations allow for a customer-focused approach and an emphasis on strategic planning and dissemination. Health communication, risk communication, and health promotion provide a theoretical and practical basis for message development, design, and delivery. Health marketing also draws on such diverse disciplines as relationship management; social marketing; mass and speech communication; public affairs and journalism; health education; instructional design; sociology and psychology; and the creation of audio, video, and multimedia products, among others. Almost all of the aforementioned health marketing foundations emphasize the fundamental importance of audience engagement, a creative aesthetic, and extensive formative evaluation.

## Improving Health Through Marketing

In this issue of *Preventing Chronic Disease*, Maibach et al (2) make a compelling case for the important role that marketing can play in public health and clearly define key constructs of marketing. The authors suggest that core marketing activities (i.e., conducting customer research, building sustainable distribution channels, and improving access to easily adopted programs) can enhance the adoption and implementation of health behaviors and practices, specifically, evidence-based prevention strategies. Furthermore, they advocate that the effective marketing of evidence-based health programs can help close the gap that exists between public health research and everyday practice.

Maibach et al are on the forefront of a growing trend in health promotion and protection. Although marketing strategies are not new to public health, their diffusion and adoption are beginning to increase in many sectors, particularly as the relationships and collaborations grow stronger between businesses and private and governmental public health organizations. CDC's health marketing efforts may provide a useful example for the development, advancement, and operationalization of other health marketing programs.

The National Center for Health Marketing (NCHM), one of the newest among 12 national centers at CDC, was established in 2004 as a result of an agencywide strategic planning process known as the Futures Initiative. The mission of NCHM is to protect and promote health and advance CDC's goals through innovative health marketing programs, products, and services that are consumer centered, high impact, and science based. All of NCHM's people and programs are committed to being customer centered by identifying and meeting the needs of our audiences and partners; being high impact by leveraging our assets, strengths, and partnerships for maximum health impact; and being science based by using and generating scientific evidence and established best practices.

NCHM's activities and capacities fall into four functional categories of marketing. First, we have a unit focused on *product design, research, and development,* with our primary product being CDC's science-based health information and messages. This group includes professionals in health communication, risk and crisis communication, market research and evaluation, marketing strategy, behavioral science, and our newest area consisting of multilingual translation, cultural communication, and health literacy activities. Maibach et al note the importance of customer research to effective marketing programs, and this NCHM unit has expertise in all aspects of customer and market research, as well as access to audience-specific databases and resources useful for developing messages and campaigns. Working with subject matter and communication experts throughout CDC, this group has extensive experience developing urgent and planned health messages and campaigns on almost any health issue for diverse audiences throughout the United States and the world.

Second, NCHM has a unit focused on *product production and packaging*, and its primary products are health information and messages. This group includes professionals in graphic art and design; audio, video, and multimedia production; animation; photography; Web development; broadcast engineering; and instructional design and production experts in other related fields. The group also has access to state-of-the art design, media, and production technology through the Global Communications Center that recently opened on the CDC campus in Atlanta, Ga.

Third, we have a unit focused on *product distribution*, which contains many of CDC's external communication channels through which health information and message products are disseminated and delivered. Maibach et al note that marketing and distribution channels are generally considered to be the most important element in the marketing mix, and CDC is fortunate to have many powerful channels for communicating with health professionals and partners, including the *Morbidity and Mortality Weekly Report (MMWR),* the *Guide to Community Preventive Services,* the Epidemic Information Exchange (Epi-X), the Health Alert Network (HAN), a professional conference exhibit booth, and many others. CDC's channels for communicating with the public include CDC's Web site (www.cdc.gov), the CDC hotline (800-CDC-INFO), the Global Communications Center gallery and exhibits, the forthcoming HHS-TV channel on the Dish Network, and many others. Together, we have a powerful array of distribution channels with which our customers can easily access and implement our products and programs.

Finally, we have a unit focused on *customer relationship management,* which coordinates CDC's new and ongoing relationships with our external partners. The active input from and relationships with our partners are one of CDC's greatest strengths. This unit works with collaborators throughout CDC to make our partner relationships as strong and as engaging as possible. As CDC's director, Dr Julie Gerberding, has noted, "CDC's partners, current and future, are critical to achieving our public health goals and to delivering our products."

As Maibach et al explain, partners can also serve as a "powerful and sustainable distribution channel." In addition to relationship management, we work closely with our partners to encourage the public distribution and amplification of CDC's health information and to ensure greater alignment and consistency of health messages, particularly during crises or emergencies. Maibach et al also note the importance of engaging all sectors of society, including emerging public health partners such as for-profit businesses. NCHM actively works with all partnership sectors, including public health systems, health care organizations, education institutions and groups, and faith and community organizations, and has recently embarked on projects to form several new partnerships and products to serve for-profit businesses.

## Conclusion

Through our health marketing work, we strive to achieve our vision: a world in which all people actively use accessible, accurate, relevant, and timely health information and interventions to protect and promote their health and the health of their families and communities. Like the application of marketing to public health, NCHM is in its early days. More research, evaluation, and accumulated experience are required to effectively translate and apply the principles of marketing to public health.

At the end of their article, Maibach et al state that significant public health resources, including training, should focus on health marketing strategies and that NCHM should cultivate these opportunities to blend marketing and public health. We accept our leadership role in this endeavor. We call on our colleagues and partners in public health and health marketing at all levels from all sectors to become involved and advocate for the advancement and proliferation of this discipline and perspective. Through our collaborative efforts, the blank stares, head shakes, and looks of confusion will turn to knowing, affirmative nods and smiles of recognition and understanding. We thank Maibach et al for drawing attention to marketing's usefulness and potential in advancing the field of public health.
